# *In vivo* monoubiquitination of anaplerotic phosphoenolpyruvate carboxylase occurs at Lys624 in germinating sorghum seeds

**DOI:** 10.1093/jxb/ert386

**Published:** 2013-11-28

**Authors:** Isabel Ruiz-Ballesta, Ana-Belén Feria, Hong Ni, Yi-Min She, William Charles Plaxton, Cristina Echevarría

**Affiliations:** ^1^Departamento de Biología Vegetal, Facultad de Biología, Universidad de Sevilla, Avda Reina Mercedes no. 6, 41012 Sevilla, Spain; ^2^Shanghai Center for Plant Stress Biology, Chinese Academy of Sciences, 3888 Chenhua Road, Shanghai 201602, China; ^3^Department of Biology, Queen’s University, Kingston, Ontario K7L 3N6, Canada

**Keywords:** Development, germination, monoubiquitination, phospho*enol*pyruvate carboxylase, post-translational modification, seeds, *Sorghum bicolor*.

## Abstract

Phospho*enol*pyruvate carboxylase (PEPC; EC 4.1.1.31) is an important cytosolic regulatory enzyme that plays a pivotal role in numerous physiological processes in plants, including seed development and germination. Previous studies demonstrated the occurrence of immunoreactive PEPC polypeptides of ~110kDa and 107kDa (p110 and p107, respectively) on immunoblots of clarified extracts of germinating sorghum (*Sorghum bicolor*) seeds. In order to establish the biochemical basis for this observation, a 460kDa PEPC heterotetramer composed of an equivalent ratio of p110 and p107 subunits was purified to near homogeneity from the germinated seeds. Mass spectrometry established that p110 and p107 are both encoded by the same plant-type PEPC gene (*CP21*), but that p107 was *in vivo* monoubiquitinated at Lys624 to form p110. This residue is absolutely conserved in vascular plant PEPCs and is proximal to a PEP-binding/catalytic domain. Anti-ubiquitin IgG immunodetected p110 but not p107, whereas incubation with a deubiquitinating enzyme (USP-2 core) efficiently converted p110 into p107, while relieving the enzyme’s feedback inhibition by l-malate. Partial PEPC monoubiquitination was also detected during sorghum seed development. It is apparent that monoubiquitination at Lys624 is opposed to phosphorylation at Ser7 in terms of regulating the catalytic activity of sorghum seed PEPC. PEPC monoubiquitination is hypothesized to fine-tune anaplerotic carbon flux according to the cell’s immediate physiological requirements for tricarboxylic acid cycle intermediates needed in support of biosynthesis and carbon–nitrogen interactions.

## Introduction

Phospho*enol*pyruvate (PEP) carboxylase (PEPC; EC 4.1.1.31) catalyses the irreversible β-carboxylation of PEP to yield oxaloacetate and P_i_. The enzyme plays a central role in the initial fixation of atmospheric CO_2_ during C_4_ and Crassulacean acid metabolism photosynthesis (Echevarría and Vidal, 2003). PEPC also fulfils a broad array of crucial non-photosynthetic functions, particularly the anaplerotic replenishment of tricarboxylic acid (TCA) cycle intermediates consumed during biosynthesis and nitrogen assimilation (Echevarría and Vidal, 2003; O’Leary *et al.*, 2011*a*). To achieve their diverse functions and complex regulation, vascular plant PEPCs belong to a small multigene family encoding several closely related PTPCs (plant-type PEPCs), as well as a single, distantly related BTPC (bacterial-type PEPC) (O’Leary *et al.*, 2011*a*). PTPC genes encode 100–110kDa polypeptides that typically assemble as Class-1 PEPC homotetramers.

A remarkable array of strategies has evolved to control plant PEPC activity post-translationally, including allosteric effectors (Echevarría *et al.*, 1994), covalent modification via phosphorylation or monoubiquitination, and protein–protein interactions (Uhrig *et al.*, 2008; O’Leary *et al.*, 2011*a*, b). PTPC phosphorylation at its conserved N-terminal seryl residue has been widely studied, and is catalysed by a dedicated calcium-independent PTPC protein kinase (PPCK). This enhances allosteric activation of Class-1 PEPCs by hexose-phosphates while reducing inhibition by l-malate and l-aspartate (O’Leary *et al.*, 2011*a*). Class-1 PEPCs were recently discovered to be subject to reciprocal control by inhibitory monoubiquitination in germinating castor oil (*Ricinus communis*) seeds (COS) (Uhrig *et al.*, 2008), as well as in immature proteoid roots of harsh hakea (*Hakea prostrata*) (Shane *et al.*, 2013). Extracts of germinating COS or immature proteoid roots of harsh hakea contain an equivalent ratio of immunoreactive PTPC polypeptides having molecular masses of ~110kDa and 107kDa (p110 and p107, respectively) (Uhrig *et al.*, 2008; Shane *et al.*, 2013). PEPC purification and mass spectrometry (MS) revealed that: (i) both subunits arise from the same *PTPC* gene and interact to form a p110:p107 heterotetrameric Class-1 PEPC; and (ii) p110 is the monoubiquitinated form of p107 (Uhrig *et al.*, 2008; Shane *et al.*, 2013). Monoubiquitination of COS or harsh hakea Class-1 PEPC is inhibitory as it increased their *K*_m_(PEP) values while enhancing sensitivity to allosteric inhibitors. These studies have provided a new paradigm for the post-translational control of non-photosynthetic Class-1 PEPCs, as well as the first examples of regulatory monoubiquitination of a metabolic enzyme in nature. Similarly, a deubiquitinating enzyme, ataxin 3, is activated by monoubiquitination (Todi *et al.*, 2009).

An immunoreactive PTPC ‘doublet’ highly reminiscent of partially monoubiquinated COS or harsh hakea PEPC has frequently been observed on immunoblots of clarified extracts from diverse plant tissues including leaves, guard cells, roots, and fruits (Denecke *et al.*, 1993; Law and Plaxton, 1995; De Nisi and Zocchi, 2000; Rao *et al.*, 2006), and during the development or germination of cereal seeds such as barley, wheat, and sorghum (Osuna et al., 1996, 1999; Gonzalez *et al.*, 1998; Nhiri *et al.*, 2000; Feria *et al.*, 2008). The overall aim of the present study was to test the hypothesis that Class-1 PEPC of germinating sorghum seeds is subject to *in vivo* monoubiquitination. The main PEPC isozyme of germinating sorghum was identified as the product of a *PTPC* gene (*CP21*) and it was established that it exists as a heterotetramer comprised of an equivalent ratio of monoubiquitinated p110 and non-ubiquitinated p107 PTPC subunits. Detailed MS analyses demonstrated that monoubiquitination of the p110 subunit occurs at a conserved Lys624 residue that aligns with the Lys628 monoubiquitination site of Class-1 PEPC from germinated COS (Uhrig *et al.*, 2008). Partial PEPC monoubiquitination was also shown to occur during sorghum seed development.

## Materials and methods

### Plant material

Sorghum [*Sorghum bicolor* (L.) Moench, var. PR87G57, Pioneer Hi-Bred Spain] seeds were sterilized and germinated as previously reported (Nhiri *et al.*, 2000). Harvested seeds were frozen in liquid N_2_ and stored at –80 ºC until used. Sorghum plants were cultivated in a greenhouse under a 14h day (25 ºC)/10h night (18 ºC) cycle. Seeds were harvested at various stages of development, ground to a powder under liquid N_2_, lyophilized, and kept at 4 ºC for later analyses.

### Antibodies

Polyclonal antibodies against: (i) native C_4_-photosynthetic PTPC from sorghum leaves (anti-C_4_ PTPC) were prepared as described in Pacquit *et al.* (1995); and (ii) synthetic peptides corresponding to the C-terminal [(Y) EDTLILTMKGIAAGMQNTG] and dephosphorylated N-terminal [ERHHSIDAQLRALAPGKVSEE24(YG)] ends of C_4_-photosynthetic PTPC (anti-C19 and anti-N24, respectively), and the SIDAQLR phosphorylation motif (anti-SIDAQLR) common to all known PTPC sequences were from NEOSYSTEM S.A. (Strasburg, France). Anti-pSer13-IgG (anti-pSer13) was raised against a synthetic phosphopeptide corresponding to the N-terminal seryl phosphorylation site of a sorghum C_3_ PTPC sequence [phosphorylated on the regulatory serine: Cys-ERLS(**pS**)IDAQLR] (Lepiniec *et al.*, 1993). Anti-COS PTPC-IgG (anti-COS PTPC) was raised against native Class-1 PEPC of developing COS as described in Tripodi *et al.* (2005). Anti-ubiquitin IgG (anti-UB) was purchased from Millipore, Canada (catalogue no. 05-944).

### Protein extraction

Frozen powder (0.4g) from germinated sorghum seeds was ground in a chilled mortar with sand in an ice-cold buffer which contained 1ml of 100mM TRIS-HCl (pH 7.5), 10mM MgCl_2_, 1mM EDTA, 20% (v/v) glycerol, 14mM β-mercaptoethanol, 1mM phenylmethylsulphonyl fluoride, 10 μg ml^–1^ chymostatin, 10 μM leupeptin, 50mM KF, 1mM Na_2_MoO_4_, 1mM Na_3_VO_–4_, and 50nM microcystin-LR or okadaic acid. Homogenates were centrifuged at 17 000 *g* for 7min. Clarified extract proteins were precipitated by the addition of 60% (saturation) (NH_4_)_2_SO_4_, pelleted by centrifugation at 10 500 *g*, resuspended in 200 μl of extraction buffer, and desalted by chromatography on a Sephadex G-25 column (1×5cm) that had been equilibrated in extraction buffer lacking β-mercaptoethanol.

### Electrophoresis and immunoblotting

Samples were subjected to SDS–PAGE [7.5% (w/v) acrylamide] and immunoblotting as previously described (Tripodi *et al.*, 2005). Antigenic polypeptides were visualized using an alkaline phosphatase-conjugated secondary antibody with chromogenic detection (Tripodi *et al.*, 2005). Alternatively, immunolabelled proteins were detected by a chemiluminescence detection system (SuperSignal West Pico Rabbit IgGs; Thermo Scientific) according to the manufacturer’s instructions in a Fujifilm LAS3000 mini-system.

### Determination of PEPC activity and kinetic studies

PEPC activity was assayed at 340nm using a Molecular Devices Spectramax Kinetics Microplate reader. Optimized assay conditions were: 50mM HEPES-KOH, pH 8.0, 2.5mM PEP, 5mM NaHCO_3_, 5mM MgCl_2_, 0.15mM NADH, 10% (v/v) glycerol, 1mM dithiothreitol (DTT), and 5U ml^–1^ porcine heart malate dehydrogenase (0.2ml final volume). One unit of activity is defined as the amount of PEPC catalysing the carboxylation of 1 μmol of PEP min^–1^ at 25 ºC. Protein concentrations were determined by the Coomassie Blue G-250 dye binding method using bovine γ-globulin as the standard (Tripodi *et al.*, 2005).

Apparent *K*_m_ and IC_50_ (concentration of inhibitor producing 50% inhibition) values were calculated using a computer kinetics program as previously described (Tripodi *et al.*, 2005). All kinetic parameters are the means of three independent experiments and are reproducible to within ±15% (SE) of the mean value. Stock solutions of metabolites were made equimolar with MgCl_2_ and adjusted to pH 7.5.

### Buffers used during PEPC purification

All buffers contained 50mM HEPES-KOH (pH 8.0), 5mM MgCl_2_, 1mM DTT, and a phosphatase inhibitor cocktail consisting of 5mM EDTA, 1mM EGTA, 25mM NaF, 1 mMM Na_2_MoO_4_, 1mM Na_3_VO_4_^–^, and 5mM NaPP_i_. Buffer A contained 20% (v/v) glycerol, 0.1% (v/v) Triton X-100, 1% (w/v) insoluble poly(vinylpolypyrrolidone), 1mM phenylmethylsulphonyl fluoride, and 2mM 2,2′-dipyridyl disulphide. Buffer B contained 25% (saturation) (NH_4_)_2_SO_4_. Buffer C consisted of buffer B lacking (NH_4_)_2_SO_4_ but containing 10% (v/v) ethylene glycol. Buffer D contained 20% (v/v) glycerol and 50mM KCl. Buffer E contained 20% (v/v) glycerol and 100mM KCl.

### PEPC purification

All procedures were performed at room temperature unless otherwise noted. Frozen whole 48h germinated sorghum seeds (300g) were homogenized (1:2.5; w/v) in ice-cold buffer A using a Polytron and centrifuged at 4 ºC for 20min at 10 000 *g*. The supernatant was filtered through Miracloth and brought to 25% (saturation) (NH_4_)_2_SO_4_, stirred for 20min at 4 ºC, and centrifuged as above. The supernatant was adjusted to 60% (saturation), stirred, and centrifuged as above. Pellets were resuspended in buffer B lacking (NH_4_)_2_SO_4_. The solution was loaded at 2ml min^–1^ onto a column (2.2×10cm) of butyl-Sepharose 4 Fast Flow pre-equilibrated with buffer B and connected to an ÄKTA Purifier FPLC (fast protein liquid chromatography) system (GE Healthcare). The column was washed until the *A*_280_ decreased to baseline. PEPC was eluted by 50% buffer C (50% buffer B) (6ml per fraction^–1^). Pooled peak fractions were concentrated using an AMICON Ultra-15 ultrafiltration device (30kDa cut-off) (Millipore), frozen in liquid N_2_, and stored at –80 ºC overnight. The sample was thawed and adjusted to contain 25% (w/v) polyethylene glycol (average mol. wt. 8000Da). The solution was incubated for 25min on ice and centrifuged at 18 000 *g* for 15min at 4 ºC. Pellets were resuspended in buffer D containing 50nM microcystin-LR and 2.5 μl ml^–1^ ProteCEASE-100 (G-Biosciences). The sample was loaded at 0.75ml min^–1^ onto a column (1×3cm) of Fractogel EMD DEAE-650 (S) that had been pre-equilibrated with buffer D. The column was washed with buffer D until the *A*_280_ decreased to baseline, and PEPC activity was eluted with a 50–500mM linear KCl gradient (40ml) in buffer D (2ml per fraction). Pooled peak fractions were concentrated to 0.6ml as above and centrifuged at 14 000 *g* for 5min at 4 ºC. The supernatant was brought to 1ml with buffer E and loaded at 0.40ml min^–1^ onto a calibrated Superdex-200 HR 16/60 gel filtration FPLC column that had been pre-equilibrated with buffer E. Peak activity fractions were pooled and concentrated to 0.25ml as above. The final preparation was diluted to 0.5ml with buffer E, divided into 25 μl aliquots, frozen in liquid N_2_, and stored at –80 ºC. PEPC activity remained stable for at least 3 months when stored frozen.

### Deubiquitination

Clarified seed extracts (50 μg) or the purified PEPC from germinated seeds (6 μg) were respectively incubated at 37 ºC for up to 60min with 5 μM and 20 μM ubiquitin-specific protease-2 core (USP-2) (Abcam, cat.# ab125735) in a final volume of 50 μl. The incubation buffer was 25mM TRIS-HCl (pH 7.5), 50mM KCl, 5mM MgCl_2_, 20% (v/v) glycerol, 1mM DTT, 0.5mM Na_2_MoO_4_, and 0.5mM Na_3_VO_4_^–^.

### Mass spectrometry

Coomassie Blue-stained polypeptides were excised from SDS gels, in-gel reduced, alkylated, and digested with trypsin using standard protocols (Uhrig *et al.*, 2008). Tryptic peptides were extracted using acetonitrile/0.1% trifluoroacetic acid (TFA) (v/v, 60:40), and dried by a CentriVap refrigerated centrifugal concentrator (Labconco Corp.). Peptides were reconstituted in 4 μl of 0.1% formic acid (FA) and identified using two instrumentation methods: a Eksigent nanoLC 400 system coupled with a TripleTOF 5600+ quadrupole time-of-flight (TOF) mass spectrometer (AB Sciex, Concord, ON, Canada) at low-energy collision-induced dissociation (CID); and an EASY-nLC Orbitrap Fusion Tribrid LC-MS system (Thermo Fisher, San Jose, CA, USA) at higher energy collision-induced dissociation (HCD) mode. The sample was trapped by a reverse-phase (RP) C_18_ column (Chrom XP, 350 μm id×0.5mm length, 3 μm, 120A) at 2 μl min^–1^ of solvent A (0.1% FA) for 15min, and then separated through a C_18_ analytical column (75 μm id×150mm, 3 μm, 120A) at 300 nl min^–1^ for 60min. The mobile phase was set up to a linear gradient from 5% to 30% solvent B (0.1% FA in acetonitrile), followed by 85% solvent B over 10min for peptide elution. TripleTOF MS and MS/MS scans were acquired with a high resolution of 30 000, and the *m/z* regions of the MS survey scan and MS/MS were selected from *m/z* 350–1600 and *m/z* 100–1250, respectively, and by data-dependent scanning of the top 20 ions at multiply charged states of 2+, 3+, and 4+. Dynamic exclusion was set to a period of time at 30 s. For the purpose of comparison, similar analyses were performed on the gel-separated proteins using the Orbitrap Fusion Tribrid LC-MS system under high-accuracy MS survey scan at a resolution of 120 000 followed by sequential HCD scans of the top 10 ions. MS/MS data from the two instruments were searched against the viridiplantae (green plants) protein sequences in the NCBI database using Mascot Server (version 2.4.0, Matrix Science, London, UK). The search parameters were restricted to tryptic peptides at a maximum of two missed cleavages. Cysteine carbamidomethylation was designated as a fixed modification, and deamidation of asparagine and glutamine, oxidation of methionine, phosphorylation of serine/threonine/tyrosine, and ubiquitination of lysine were considered as variable modifications. Mass tolerances were set up to 30 ppm for MS ions and 0.05Da for MS/MS fragment ions. Peptide assignments were filtered by an ion score cut-off at 15, and the identified MS/MS spectra were also verified manually.

### Statistical analysis

This was performed using statistical software (SigmaStat, Systat Software Inc., San José, CA, USA). Data were analysed using the Student’s *t-*test. Means were considered to be significantly different at *P* < 0.05.

## Results and Discussion

### Influence of sorghum seed germination on PEPC subunit structure and post-translational modifications

**Fig. 1.**
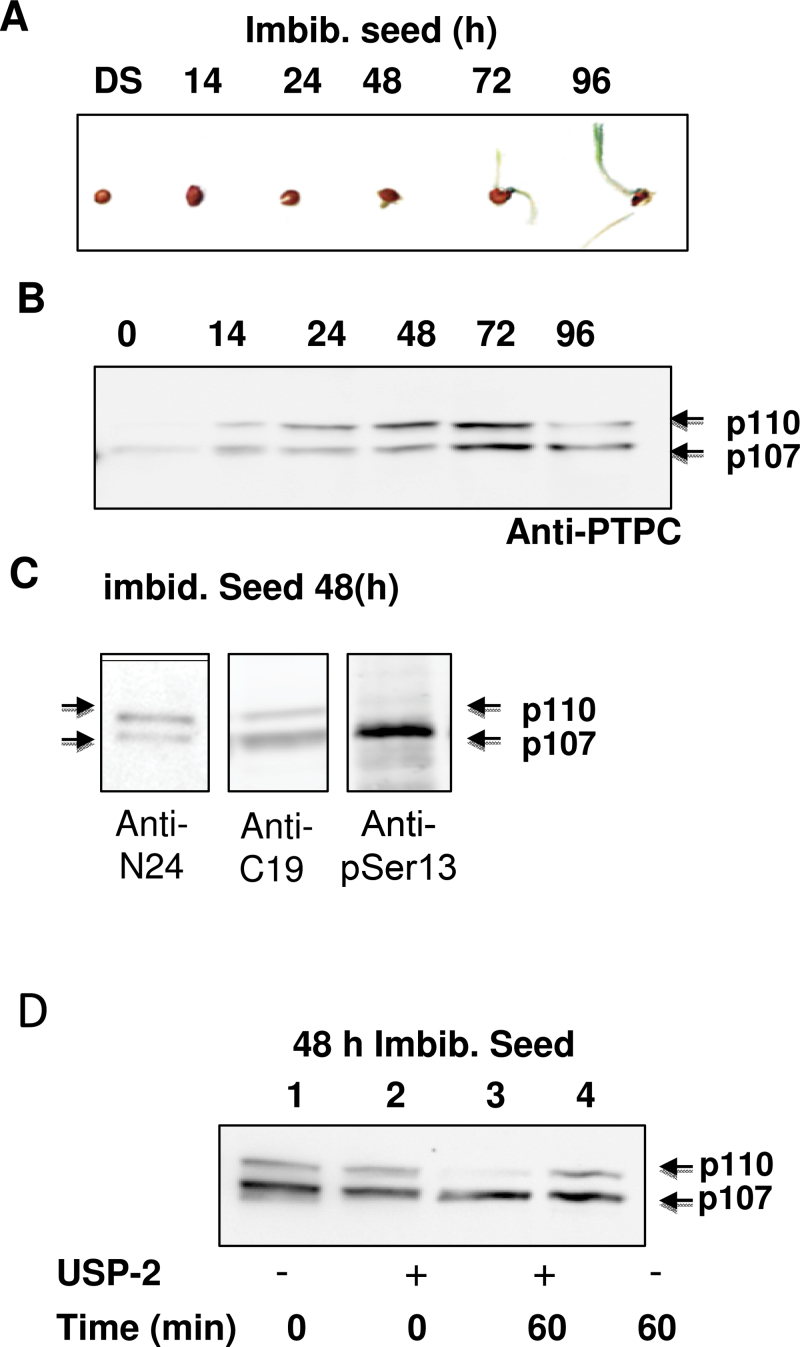
Immunocharacterization of p110 and p107 PEPC subunits during sorghum seed germination. (A) Stages of seed germination; DS, dry seed. (B) Seed extracts were subjected to SDS–PAGE (8% gel, 40 μg of protein), electroblotted onto a PVDF membrane, and probed with anti-C_4_ PEPC. (C) Immunoblots of 48h post-imbibition extracts were probed with anti-N24, anti-pSer13, and anti-C-19. (D) Monoubiquitination of p110. Clarified extracts from seeds imbibed in water for 48h were incubated in the presence or absence of 1 μM USP-2 core (Abcam) for 1h at 30 ºC, and subjected to immunoblot analysis using anti-C_4_ PEPC; 50 μg protein lane^–1^.

Preliminary immunoblot experiments corroborated an earlier report (Nhiri *et al.*, 2000) that sorghum seed germination is accompanied by: (i) accumulation of comparable amounts of pre-existing approximate 107kDa and inducible 110kDa immunoreactive PTPC polypeptides (p107 and p110, respectively) (Fig. 1B); and (ii) *in vivo* phosphorylation of p107 at its conserved N-terminal seryl residue (Fig. 1C; anti-pSer13). Both subunits are intact since the anti-C19 and anti-N24 {antibodies raised against synthetic peptides corresponding to the C-terminal [(Y) 942-EDTLILTMKGIAAGMQNTG-960] and dephosphorylated N-terminal [4-ERHHSIDAQLRALAPGKVSEE-24(YG)] ends, respectively, of C_4_-photosynthetic PTPC} both cross-reacted with p110 and p107 (Fig. 1C). Incubation with USP-2, a recombinant human deubiquitinating enzyme, resulted in p110’s apparent deubiquitination (Fig. 1D). Subsequent experiments confirmed that p110 is a monoubiquitinated form of p107 (see below). As the USP-2 core lacks an N-terminal regulatory domain, it can deubiquinate a large number of substrates *in vitro*, including monoubiquitinated Class-1 PEPCs from germinating COS and developing proteoid roots of harsh hakea (Uhrig *et al.*, 2008; Shane *et al.*, 2013).

### PEPC purification

**Fig. 2.**
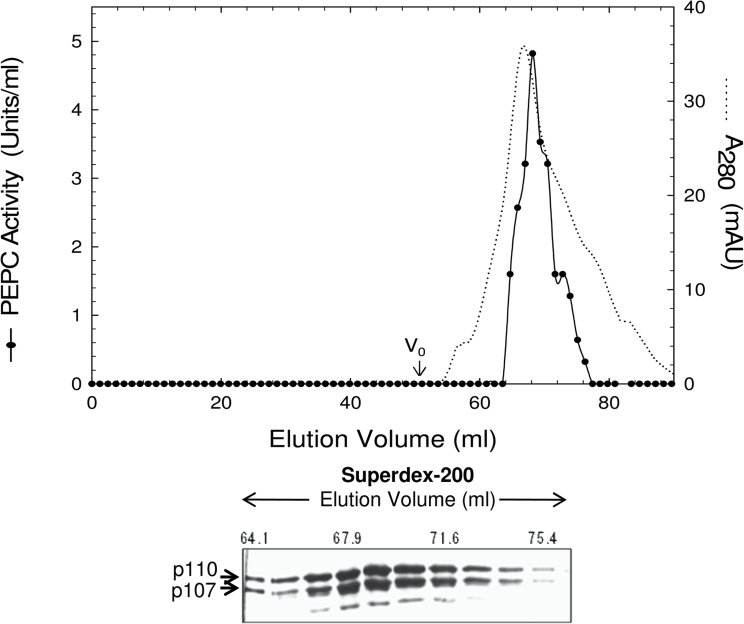
Co-elution of PEPC activity with 110kDa and 107kDa PEPC polypeptides (p110 and p107, respectively) during Superdex-200 HR 16/60 gel-filtration FPLC of PEPC from germinating sorghum seeds. Aliquots (10 μl each) from various fractions were subjected to SDS–PAGE and immunoblot analysis using anti-COS PTPC. V_0_ denotes the column’s void volume.

**Fig. 3.**
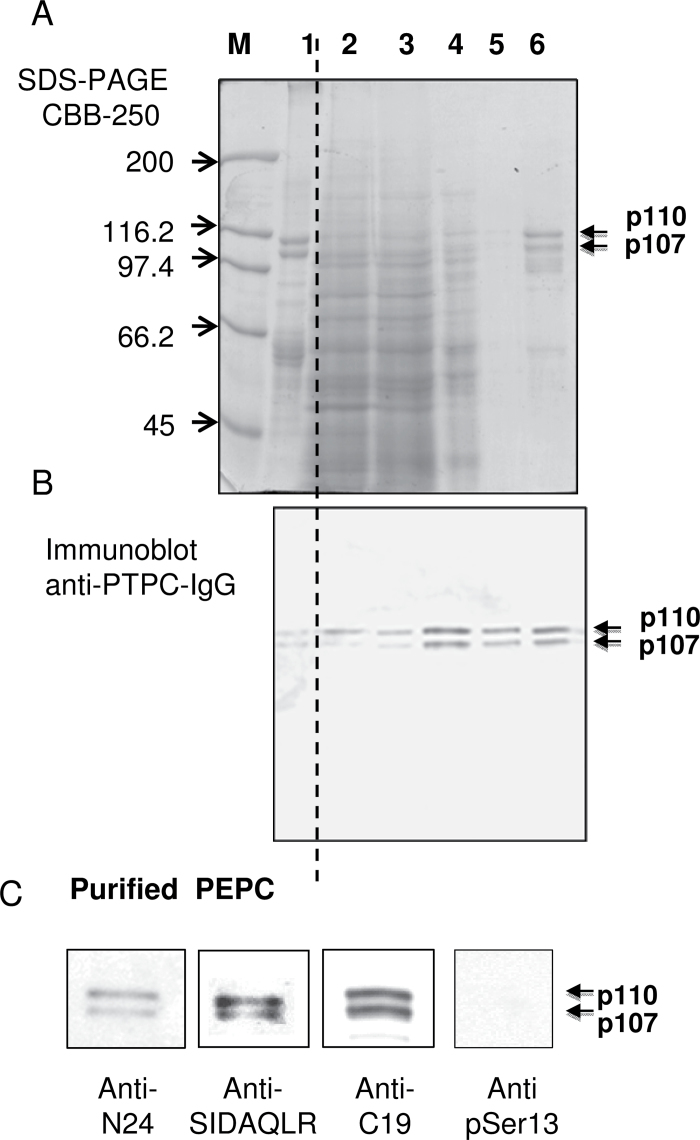
SDS–PAGE and immunoblot analysis of various fractions obtained during the purification of PEPC from 48h germinated sorghum seeds. (A) SDS–PAGE was followed by protein staining with Coomassie Blue R-250 (CBB-250). (B) Immunoblot analysis was performed using anti-COS PTPC. Lane 1, 2.5 μg (A) or 50ng (B) of purified monoubiquitinated Class-1 PEPC (RcPPC3) from germinating COS endosperm (Uhrig *et al.*, 2008). Lane 2, 45 μg (A) or 15 μg (B) of protein from the clarified extract of sorghum seeds. Lane 3, 45 μg (A) or 15 μg (B) of 25–60% (NH_4_)_2_SO_4_ fractions. Lane 4, 8 μg (A) or 4 μg (B) of the butyl-Sepharose fraction. Lane 5, 2 μg (A) or 0.1 μg (B) of the DEAE-Fractogel fraction. Lane 6, 2 μg (A) or 0.1 μg (B) of Superdex 200 fractions. ‘M’ denotes various protein molecular weight standards. (C) N-terminal immunocharacterization of the purified PEPC from 48h germinated sorghum seeds. Samples were subjected to 8% SDS–PAGE, blot-transferred onto a PVDF membrane, and probed with anti-N24, anti-SIDAQLR, anti-C19, and anti-pSer13. All lanes contain 0.6 μg of protein except lane anti-C19 that contains 0.2 μg.

**Fig. 4.**
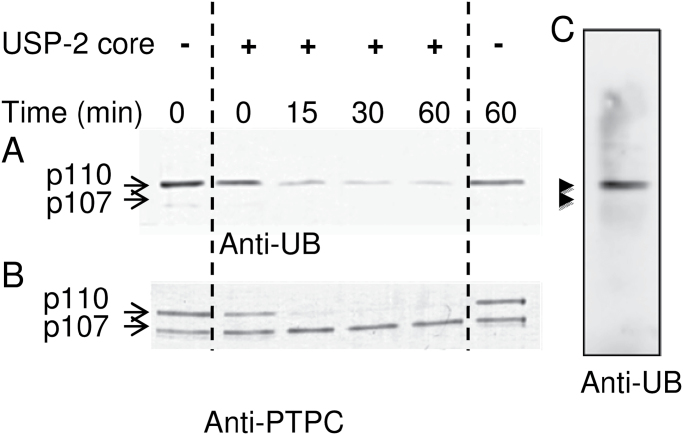
Incubation with the deubiquitinating enzyme USP-2 core converts the p110:p107 heterotetrameric PEPC from germinated sorghum seeds into a p107 homotetramer. Purified germinated sorghum seed PEPC was incubated in the presence or absence of 20 μM USP-2 core, at 30 ºC. Aliquots were removed at various times and subjected to immunoblot analysis using (A and C) anti-UB (250ng PEPC lane^–1^) or (B) anti-COS PTPC (25ng PEPC lane^–1^).

PEPC from the 48h germinated seeds was purified 391-fold to near homogeneity and an overall yield of 29% (Fig. 2A; Supplementary Table S1 available at *JXB* online). The final specific activity of 7.1U mg^–1^ compares favourably with the value of 9.6U mg^–1^ obtained with purified monoubiquitinated PEPC from germinating COS (Uhrig *et al.*, 2008). The single peak of PEPC activity resolved during Superdex-200 gel filtration FPLC co-eluted with an equivalent ratio of immunoreactive p110 and p107 (Fig. 2). Similarly, SDS–PAGE and immunoblotting indicated that the final preparation consisted of equivalent amounts of protein-staining or anti-COS-PTPC-immunoreactive p110 and p107 that respectively co-migrated with the subunits of purified Class-1 PEPC from germinated COS (Fig. 3A, B). The enzyme’s native molecular mass was estimated by analytical gel filtration to be ~460kDa (Supplementary Fig. S1). In agreement with earlier results obtained with clarified sorghum seed extracts (Fig. 1D), p110, but not p107, of the purified PEPC was immunodecorated by anti-UB (Fig. 4A); this cross-reaction was eliminated together with the protein-staining p110 band upon USP-2 treatment of the final preparation (Fig. 4B). No higher molecular mass immunoreactive polypeptides indicative of polyubiquitination were observed when the final preparation was probed with anti-UB (Fig. 4C).

The p110 and p107 subunits of Class-1 PEPC from germinated COS are both truncated by 19 amino acids such that their N-terminal phosphorylation domain is absent; this process appears to occur *in vivo* and could therefore have physiological significance (Uhrig *et al.*, 2008). In contrast, the p110 and p107 subunits of the purified sorghum seed PEPC appear to be intact since they both cross-reacted with the anti-N24, anti-SIDAQ, and anti-C19 (Fig. 3C). Despite evidence for p107 phosphorylation in clarified extracts of 48h germinated sorghum seeds (Fig. 1C; anti-pSer13) (Nhiri *et al.*, 2000), the N-terminal seryl phosphorylation site of the p110 or p107 subunits of the final preparation did not appear to be phosphorylated, as indicated by immunoblotting with anti-pSer13 (Fig. 3C) (confirmed below via MS). However, both subunits readily incorporated ^32^P when the final preparation was incubated with [γ-^32^P]ATP and recombinant sorghum PPCK2 or PPCK3 (the physiological PPCKs that catalyse *in vivo* phosphorylation of sorghum PTPC) (Supplementary Fig. S2 at *JXB* online) (Echevarría and Vidal, 2003). In addition, both subunits of the *in vitro* phosphorylated enzyme efficiently cross-reacted with anti-pSer13 (Supplementary Fig. S2). Thus, phosphorylation and monoubiquitination of the same PTPC polypeptide are not mutually exclusive, at least *in vitro*. The ^32^P autoradiography also revealed PPCK2 and PPCK3 autophosphorylation (Supplementary Fig. S2), as previously described (Monreal *et al.*, 2013). The overall results suggest that two different Class-1 PEPC populations might co-exist in germinating sorghum seeds (e.g. a monoubiquitinated dephosphorylated heterotetramer versus a phosphorylated homotetramer), or that a co-purifying phosphatase mediated *in vitro* p107 dephosphorylation during PEPC purification (despite the inclusion of a phosphatase inhibitor cocktail in all purification buffers). Further studies are needed to address the interplay between *in vivo* PTPC phosphorylation versus monoubiquitination in germinating sorghum seeds.

### Mass spectrometry

**Fig. 5.**
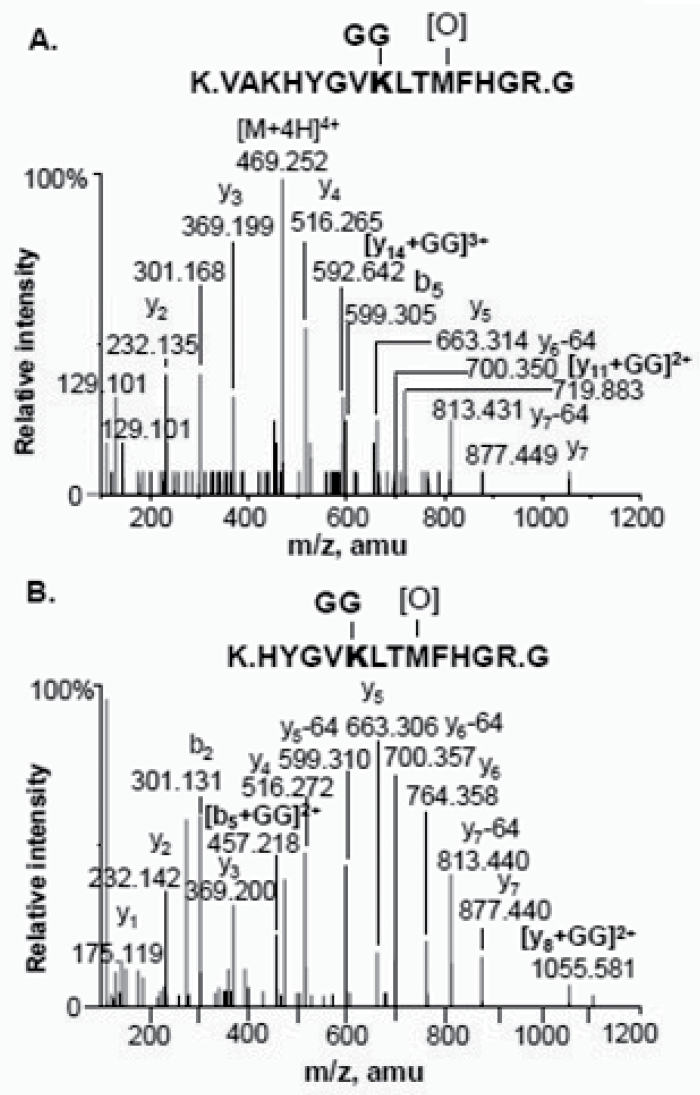
The p110 subunit of purified PEPC from germinated sorghum seeds is monoubiquitinated at Lys624. (A) TripleTOF MS/MS collision-induced dissociation (CID) analysis of the quadruply charged peptide ion of *m/z* 469.2522. (B) Orbitrap MS/MS high-energy collision-induced dissociation (HCD) analysis of the triply charged peptide ion of *m/z* 525.94. C- and N-terminal fragment ions are denoted by y and b, respectively.

Coomassie Blue R-250-stained p110 and p107 were excised from SDS gels of the final preparation and subjected individually to in-gel tryptic digestion and detailed MS analyses. Mascot analysis of p110 and p107 data sets derived from nanospray HPLC TripleTOF MS of their tryptic peptides (sequence coverage=81%) revealed that both subunits originated from the same sorghum *CP21 PTPC* gene (gi|242061132 or Sb8720). *CP21* transcripts were also induced during sorghum seed germination (Supplementary Fig. S3 at *JXB* online). *CP21* expression in both etiolated and green sorghum leaves, and also in roots depending on nitrogen supply, was previously reported (Lepiniec *et al.*, 1993). The present results establish CP21 (predicted molecular mass 109.4kDa) as the predominant PEPC isozyme expressed in germinating sorghum seeds. Consistent with earlier results: (i) p110 and p107 N-terminal tryptic peptides (residues 5–13) containing non-phosphorylated Ser7 were identified by Orbitrap LC MS/MS; and (ii) p110, but not p107, was ubiquitinated. Trypsin digestion of ubiquitin-conjugated proteins produces a signature peptide at their ubiquitination site containing a diglycine remnant derived from ubiquitin’s C-terminus and that remains attached to the target lysine residue via an isopeptide bond (Uhrig *et al.*, 2008). MS/MS analysis of the quadruply charged p110 peptide ion of *m/z* 469.2522 corresponding to residues 617–631 showed that the masses of the C-terminal fragments up to y7 have a mass change of 16Da corresponding to the oxidation of Met627, and that the ubiquitination site was localized at Lys624 based on the mass increase of 114Da (characteristic mass increment indicating glycine–glycine attachment) on the larger fragments of y11 and y14 (Fig. 5A). LC MS/MS using a high resolution Orbitrap Fusion Tribrid instrument identified a smaller ubiquitinated peptide fragment at residues 620–631 (Fig. 5B). Under high-resolution HCD fragmentation, the mass shift of 114Da on both the N-terminal b5 ion and the C-terminal y8 ion unambiguously confirmed Lys624 as p110’s monoubiquitination site. This residue precisely aligns with the Lys628 monoubiquitination site previously determined for the p110 subunit of Class-1 PEPC from germinated COS (Uhrig *et al.*, 2008). As this lysine residue appears to be absolutely conserved in all plant PTPCs, monoubiquitination at this site may be a universal PTM (post-translational modification) that occurs with the PTPC subunits of Class-1 PEPCs throughout the plant kingdom (Supplementary Fig. S4). It is also intriguing that this site is only several amino acid residues on the N-terminal side of a crucial and absolutely conserved PEP-binding/catalytic domain of all PEPCs (Supplementary Fig. S4).

### Kinetic studies

The impact of various metabolite effectors (2mM each) on the activity of the purified, monoubiquitinated heterotetrameric sorghum seed PEPC (UB-PEPC) versus the *in vitro* deubiquitinated homotetrameric enzyme (deUB-PEPC) was assessed using suboptimal assay conditions (0.1mM PEP, pH 7.3 and 8.0). With the exception of l-malate, monoubiquitination of germinating sorghum seed PEPC (CP21) exerted little to no influence in the response to the metabolites tested (Supplementary Table S2 at *JXB* online). UB-PEPC displayed a significantly lower IC_50_ for l-malate relative to deUB-PEPC (1.8mM and 3.6mM, respectively) (Table 1). In addition, the *K*_m_(PEP) value of UB-PEPC appeared to be slightly higher than that of deUB-PEPC, although the differences were not statistically significant (Table 1). Similarly, monoubiquitination of COS and harsh hakea Class-1 PEPCs is inhibitory, as it enhances sensitivity to allosteric inhibitors such as l-malate and l-aspartate, while interfering with the enzyme’s ability to bind PEP (Uhrig *et al.*, 2008; Shane *et al.*, 2013).

**Table 1.** Influence of USP-2-mediated deubiquitination on allosteric effector sensitivity of purified PEPCDeUB-PEPC was prepared by incubating UB-PEPC for 1h with 20 μM USP-2 as described in the Materials and Methods. IC_50_ values were determined using subsaturating 0.1mM PEP at pH 8.0 and/or pH 7.3.

Kinetic parameter	pH 7.3		pH 8.0	
	UB-PEPC	DeUB-PEPC	UB-PEPC	DeUB-PEPC
*K*_m_(PEP)	0.039±0.002	0.033±0.003	0.044±0.004	0.036±0.005
IC_50_(malate)	1.81±0.13*	3.62±0.24*	ND	ND
IC_50_(isocitrate)	1.47±0.17	1.37±0.12	ND	ND

All values are given in mM and represent the means ±SE of *n*=3 independent experiments. The asterisk indicates statistically significant differences between DeUB-PEPC and UB-PEPC (*P* < 0.05).

ND, not determined.

### Class-1 PEPC monoubiquitination also occurs in developing sorghum seeds

**Fig. 6.**
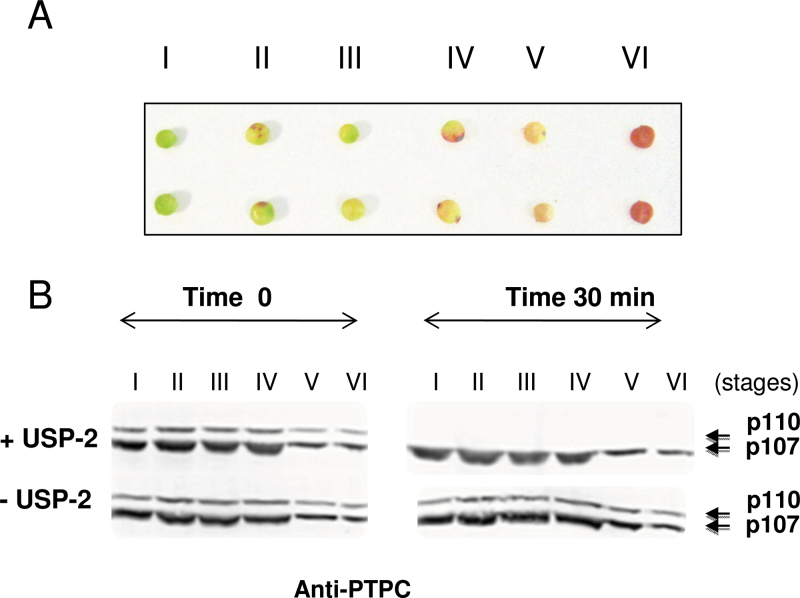
PEPC monoubiquitination in developing sorghum seeds. (A), Developmental stages of seed considered in this work, I (4–12), II (13–15), III (16–20), IV (21–27), V (28–30), and VI (31–40) days post-anthesis. (B) Clarified extracts from developing seeds were incubated in the presence and absence of 5 μM USP-2 core for 30min and subjected to immunoblot analysis using anti-COS PTPC (45 μg protein lane^–1^).

The Class-1 PEPC of developing COS endosperm exists as a 410kDa homotetramer composed of identical, *in vivo* phosphorylated p107 subunits, and also as a novel 910kDa Class-2 PEPC hetero-octameric complex in which the same Class-1 PEPC isozyme (RcPPC3) is tightly associated with four BTPC (RcPPC4) subunits (Tripodi *et al.*, 2005; Gennidakis *et al.*, 2007; O’Leary *et al.*, 2011*a*). However, neither Class-1 nor Class-2 PEPC of intact, developing COS is monoubiquitinated (O’Leary *et al.*, 2011*a*, *b*). In the cereal seeds wheat, barley, and sorghum, Class-1 PEPC has been defined as a phosphorylated enzyme in the middle of the developmental stages, dephosphorylated in dry seed, and then re-phosphorylated during germination (Osuna et al., 1996, 1999; González *et al.*, 1998; Nhiri *et al.*, 2000; Feria *et al.*, 2008). The presence of a Class-2 PEPC complex containing tightly associated PTPC and BTPC subunits has not yet been established in barley or sorghum seeds (Feria *et al.*, 2008, and unpublished data, respectively), indicating clear differences in the PEPC biochemistry of developing castor (oil-storing) versus cereal (starch-storing) seeds. It was also of interest to explore whether PEPC monoubiquitination occurs during sorghum seed development. Immunoblot analysis of PEPC in clarified extracts prepared from whole seeds harvested at different stages post-anthesis revealed the presence of anti-COS PTPC-immunoreactive p110 and p107 subunits similar to those occurring in the germinating seeds. Incubation with USP-2 led to the disappearance of immunoreactive p110 at each stage of development (Fig. 6B, +USP-2). This indicates that p110 is also a monoubiquitinated version of p107 in developing sorghum seeds. This is the first report of PEPC monoubiquitination during seed development. However, elimination of photosynthate supply to developing COS by excision of intact fruit clusters caused the Class-1 PEPC’s subunits to be rapidly dephosphorylated, and then subsequently monoubiquitinated *in vivo* (O’Leary *et al.*, 2011*b*).

### Concluding remarks

It is notable that a recent survey of photosynthetic and non-photosynthetic tissues of the castor plant unexpectedly revealed that monoubiquitination, rather than phosphorylation, was the most prevalent PTM of Class-1 PEPC’s PTPC subunits that occurs in unstressed plants (O’Leary *et al.*, 2011*b*). C_4_ and Crassulacean acid metabolism photosynthetic PTPCs (including that of sorghum) also contain a lysine residue corresponding to Lys624 and Lys628 of the p110 subunit of germinating sorghum and COS PEPCs, respectively (Supplementary Fig. S4 at *JXB* online). However, it has not yet been established whether the PTPC subunits of C_4_ or Crassulacean acid metabolism photosynthetic Class-1 PEPCs are also subject to *in vivo* monoubiquitination, in addition to their diurnal control by reversible phosphorylation.

The results of the current and previous studies extend the occurrence of *in vivo* PTPC activation and inhibition by phosphorylation and monoubiquitination, respectively, to starch-storing cereal seed Class-1 PEPCs during seed germination and development. Further experiments are necessary to establish how monoubiquitination and phosphorylation combine to fine-tune anaplerotic carbon flux according to cellular demands for C_4_–C_6_ carboxylic acids in developing versus germinating seeds. However, the inhibitory influence of monoubiquitination on the kinetic properties of sorghum (Table 1), COS, or harsh hakea Class-1 PEPCs is not dramatic (Uhrig *et al.*, 2008; Shane *et al*, 2013), suggesting that this PTM may have an additional role, *in vivo*. Many studies of protein monoubiquitination in yeast and animal cells have revealed that this PTM typically functions to recruit client proteins containing a ubiquitin-binding domain. This mediates protein–protein interactions and protein localization to control diverse processes including endocytosis, protein kinase phosphorylation ‘cascades’, gene expression, and DNA repair and replication (Sadowski and Suryadinata, 2012). Thus, future studies need to assess potential ubiquitin-binding domain proteins that might interact with monoubiquitinated Class-1 PEPCs of vascular plants, as well as the possible influence of this PTM on the enzyme’s subcellular localization. Equally important will be to identify the specific E3 ligase isozyme and upstream signalling pathways that lead to widespread monoubiquitination of non-photosynthetic PTPCs *in planta*, as well as how this PTM is coordinated with PPCK-mediated phosphorylation–activation of PTPCs at their conserved N-terminal seryl residue.

## Supplementary data

Supplementary data are available at *JXB* online.

Figure S1. Native molecular mass estimation for PEPC from 2-day germinated sorghum seeds.

Figure S2. The p110 and p107 subunits of purified Class-1 PEPC from germinated sorghum seeds can be phosphorylated *in vitro* by recombinant PPCK2 and PPCK3.

Figure S3. Quantitative PCR (qPCR) analysis of *CP21* transcript levels in germinating sorghum seeds.

Figure S4. Select amino acid sequence alignment of PEPC from different sources to show the phosphorylation and monoubiquitination sites.

Table S1. Purification of PEPC from 300g of 2-day-old germinated sorghum seeds.

Table S2. Influence of various metabolites on the activity of monoubiquitinated and *in vitro* deubiquitinated PEPC purified from germinated sorghum seeds.

Supplementary Data

## Abbreviations:

BTPC: bacterial-type phospho*enol*pyruvate carboxylase
COS: castor oil seed
deUB: deubiquitinated
DTT: dithiothreitol
FPLC: fast protein liquid chromatography
Glc-6-P: glucose-6-phosphate
MS: mass spectrometry
MS/MS: tandem mass spectrometry
p110: 110kDa polypeptide
p107: 107kDa polypeptide
PEP: phospho*enol*pyruvate
PEPC: PEP carboxylase
PTPC: plant-type phospho*enol*pyruvate carboxylase
PTM: post-translational modification
UB: ubiquitin
USP: ubiquitin-specific protease.
