# Supplementary material for: In vivo monoubiquitination of anaplerotic phosphoenolpyruvate carboxylase occurs at Lys624 in germinating sorghum seeds
Source: J Exp Bot. 2013 Nov 28;65(2):443–51. doi: 10.1093/jxb/ert386 (PMC3904705; doi:10.1093/jxb/ert386)
Supplement: Supplementary Data [file supp_65_2_443__index.html]

 In vivo monoubiquitination of anaplerotic phosphoenolpyruvate carboxylase occurs at Lys624 in germinating sorghum seeds — In vivo monoubiquitination of anaplerotic phosphoenolpyruvate carboxylase occurs at Lys624 in germinating sorghum seeds — Supplementary Data 

# *In vivo* monoubiquitination of anaplerotic phosphoenolpyruvate carboxylase occurs at Lys624 in germinating sorghum seeds

## Supplementary Data

Data files

**Files in this Data Supplement:**

- Supplementary Data - Supplementary Data
