# Supplementary material for: In vivo monoubiquitination of anaplerotic phosphoenolpyruvate carboxylase occurs at Lys624 in germinating sorghum seeds
Source: J Exp Bot. 2013 Nov 28;65(2):443–51. doi: 10.1093/jxb/ert386 (PMC3904705; doi:10.1093/jxb/ert386)
Supplement: Supplementary Data [file supp_ert386_jexbot103259_file001.pdf]

Journal Experimental Botany

In vivo monoubiquitination of anaplerotic phosphoenolpyruvate carboxylase occurs at lysine-624 in germinating sorghum seeds

Isabel Ruiz-Ballesta[1], Ana-Belén Feria[1], Hong Ni[2], Yi-Min She[2], William Charles Plaxton[3] & Cristina Echevarría[1,*]

Supplementary Materials and methods

RNA Extraction and cDNA synthesis

Total RNA was isolated from 100 mg of frozen, powdered half-embryonated seeds using the IQeasy™ Plant RNA Extraction (Intron Biotechnology). Extracted nucleic acids were DNase treated to eliminate genomic DNA. RNA concentrations were determined using a NanoDrop 2000 spectrophotometer (Thermo Scientific). Reverse-transcription reactions were performed using 1 µg of purified total RNA, 1 µL ImProm-IITM Reverse Transcriptase (Promega) and a reaction buffer containing 0.5 mM dNTP, 6 mM $MgCl_2$, 20 units recombinant RNasin® ribonuclease inhibitor, and 0.5 µg oligo(dt)15.

qPCR

Quantitative polymerase chain reactions (qPCR) were performed in a final volume of 20 µL consisting of 1 µL of the cDNA, 10 µL of SensiFAST SYBR No-ROX kit (Roche) and 15 µM of the specific primers pairs as follows: *CP21* (forward 5' –TGTTGAACAGTTTCTGGAACCTCTT-3', reverse 5'-GCTTCACAAGGGC AAGCCCAAAG-3') and *18 S rRNA* (forward 5'-GGGGAAACTTACCA GGTCCA-3', reverse 5'-GGATGGCTCCGCATAGCTA-3'). PCR was conducted on the MiniOpticonTM Real-Time PCR Detection System (Biorad), and the threshold cycles (Ct) were determined using Opticon MonitorTM analysis software for all treatments. To normalize the obtained values, 18S RNA was used as internal control in each sample.

Estimation of Native Molecular Mass via Superdex 200 Gel Filtration

Native molecular-mass was calculated from a plot of K_{av} (partition coefficient) against log molecular-mass using the following protein standards: thyroglobulin

(669-kD), ferritin (440-kD), catalase (232-kD), aldolase (158-kD), bovine serum albumin (66-kD), and carbonic anhydrase (29-kD). Blue dextran was used to determine the column's void volume.

In Vitro Phosphorylation

In vitro Phosphorylation using purified PEPC (10 µg) was performed in a reaction medium containing 100 mM Tris-HCl pH 8, 10 mM $MgCl_2$, 0.25 mM P1P5-di(adenosine-5')-pentaphosphate (adenylate kinase inhibitor), 1 mM EGTA, 1 mM KF and the recombinant PEPCase-k proteins: PEPCase-k2 and PEPCase-k3 (GenBank accession numbers: XM_002452431, XM_002446745) from Sorghum bicolor L. leaves that were produced using the Gateway technology (Monreal et al., 2013). The phosphorylation reaction was initiated by the addition 1 µCi of [γ^{32}P]-ATP (10 Ci•$mmol^{-1}$) and incubated at 30 ºC for 30 min. The reaction was stopped by boiling the samples for 3 min at 90 ºC in the presence of dissociation buffer (100 mM Tris-HCl, pH 8, 25 % glycerol, 1 % SDS, 10 % β-mercaptoethanol, and 0.05 % bromophenol blue, by vol.). The denatured proteins were separated by SDS-PAGE in a Miniprotean electrophoresis cell (Bio-Rad) and stained with Coomassie Brilliant Blue R-250. The gel was analyzed with a phosphor imager (Fujix BAS 1000; Fuji).

Sequence Alignment

Multiple alignment of full-length PEPC sequences from different plant and bacterial sources was performed using the BioEdit Sequence Alignment Editor and the Mega version 5.10 program aligning by ClustalW. The NCBI accesion numbers of the sequences used are as follows: *Ricinus_communis* RcPPC3 (ABR2987), *Sorghum bicolor* 8720-CP21 (XP_002451855.1), *Sorghum bicolor* 1090-CP28 (EER98700.1), *Sorghum bicolor* 4960 (EES13699.1), *Sorghum bicolor* 5090 (EES03641.1), *Sorghum bicolor* C_4 type (XP_002438521.1), *Arabidopsis thaliana* AtPPC1 (NP_175738.1), *Arabidopsis thaliana* AtPPC2 (AEC10145.1), *Arabidopsis thaliana* AtPPC3 (AEE75592.1), *Oryza sativa* Japonica Group OsPPC1 (AAG00180.1), *Zea mays* (CAD60555.1), *Glycine max* (NP_001241357.1), *Mesembryanthemum crystallinum* (CAA32728.2), *Oryza sativa* Japonica Group bacterial type (BAF03713.1), *Sorghum bicolor* bacterial type (XP_002455324.1), *Arabidopsis thaliana* AtPPC4 (AEE34835.1),

Ricinus_communis bacterial-type *RcPPC4* (ABR29877.1), *Synechocystis sp*. PCC 6803(BAA18393.1), *Escherichia coli* (ZP_03034212).

Supplementary Tables

Supplementary Table S1. Purification of PEPC from 300 g of 2-day-old germinated sorghum seeds.

Step	Volume	Activity	Protein	Specific activity	Purificat ion	yield
	mL	*units*	*mg*	*units* mg^{-1}	*-fold*	*%*
Clarified extract	600.0	82.0	4486	0.018	1	100
$(NH_4)_2SO_4$ Fractionation	160.0	53.5	1888	0.028	2	65
Butyl-Sepharose	22.0	99.0	186	0.53	29	121
PEG Precipitation	16.0	51.2	120.0	0.43	23	62
DEAE-Fractogel	1.0	35.0	5.0	7.0	383	43
Superdex 200	0.4	24.0	3.4	7.1	391	29

Supplementary Table S2. Influence of various metabolites on the activity of ubiquitinated and *in vitro* deubiquitinated PEPC purified from germinated sorghum seeds.

Assays were conducted at pH 8.0 and/or pH 7.3 using a subsaturating PEP 0.1 mM. Deubiquitinated PEPC (DeUB-PEPC) was prepared by incubating ubiquitinated PEPC (UB-PEPC) for 1 h with 20 µM USP-2 as described in the Materials and Methods. PEPC activity in the presence of 2 mM of each effector is expressed relative to the control set at 100%. All values represent means of three independent experiments and are reproducible to within ± 10% S.E.M of the mean value.

Addition	Relative activity			
	pH 7.3		pH 8.0	
	DeUB-PEPC	UB-PEPC	DeUB-PEPC	UB-PEPC
Glc-6-P	112	127	120	121
Glc-1-P	111	115	116	116
Fru-1-P	113	116	116	116
Ribose-5-P	98	98	105	105
Glycerol-3-P	108	107	120	124
Malate	**67**	**53**	93	93
Isocitrate	47	47	38	38
Aspartate	91	94	109	109
ATP	88	90	111	100

Supplementary figures

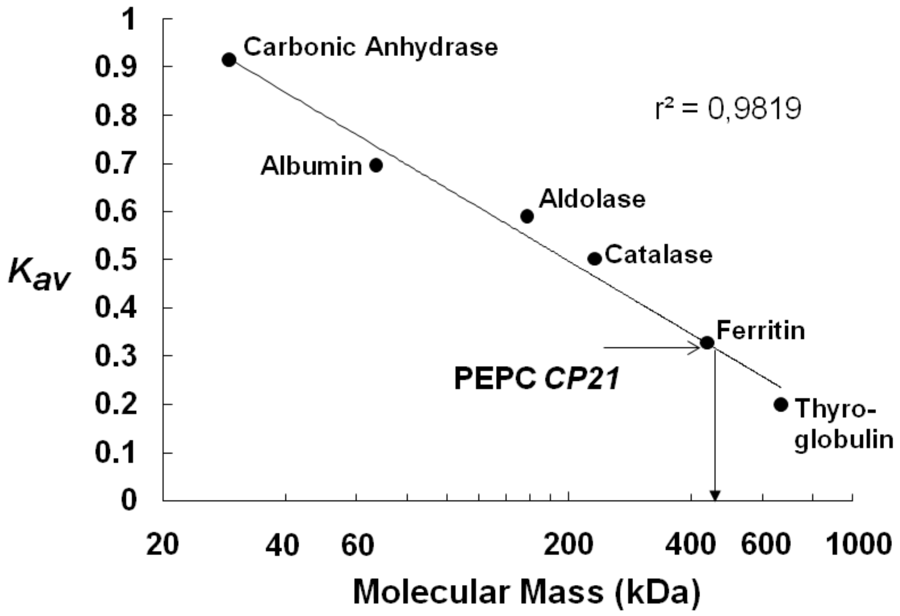

Supplementary Figure S1. Native molecular-mass estimation for PEPC from 2-day germinated sorghum seeds. This was performed during FPLC on a calibrated Superdex-200 HR 16/60 gel-filtration column as described in the Materials and Methods. r^2, correlation coefficient

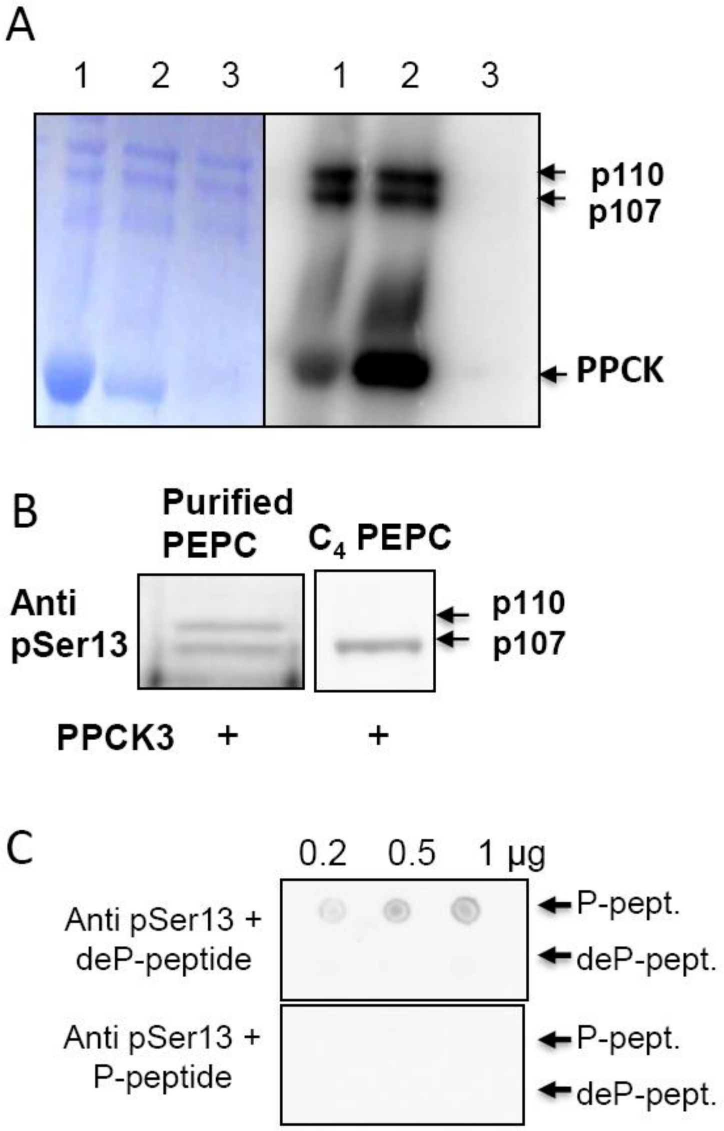

Supplementary Figure S2: The p110 and p107 subunits of purified Class-1 PEPC from germinated sorghum seeds can be phosphoryated in vitro by recombinant PPCK2 and PPCK3. (A) The purifed PEPC (10 µg) was incubated at 30 ºC with PPCK2 (lane 1), PPCK3 (lane 2) or without kinase (lane 3) in presence of 1 µCi of [γ32P]-ATP (10 Ci•mmol^{-1}). The gels were stained with Coomassie Brilliant Blue R-250 (left image) and autoradiographed (right image) using a phosphor imager. (B), Experiment as in (A) but revealed with anti pSer13. (C), Specificity of anti pSer13 antibodies.

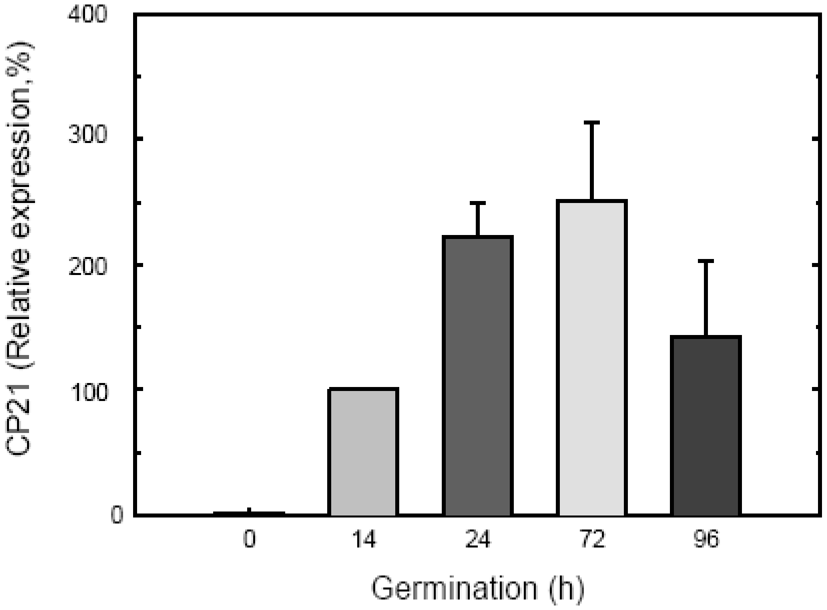

Supplementary Figure S3. Quantitative PCR (qPCR) analysis of the *CP21* transcript levels in embryonated seed halves. To normalize the values, *18S RNA* was used as internal control in each sample. Error bars represents error standard of three biological replicates. The Ct values (mean ± S.E, n = 3) were: *18S RNA* = 7.98 ±0.6; *CP21* = 23.04 ±0.6.

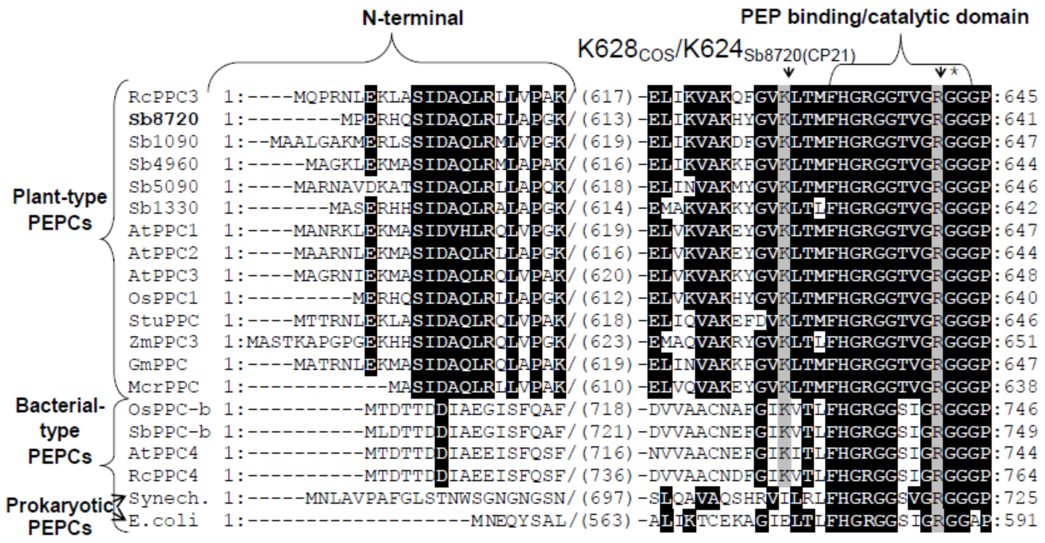

Supplementary Figure S4. Select amino acid sequence alignment of PEPC from different sources to show the phosphorylation and monoubiquitination sites. The arrow denotes the Lys-624 and Lys-628 monoubiquitination sites of p110 subunits of Class-1 PEPC from germinating sorghum seeds and COS (Uhrig et al., 2008), respectively, that is conserved in all PTPC and BTPC sequences and is highlighted by grey shading. This site is immediately adjacent to conserved catalytic domain (PEP binding site; highlighted by grey shading). This provides a logical rationale as to why monoubiquitination interferes with PEP binding to the enzyme (e.g. steric hinderance caused by UB molecule).The accession numbers of these sequences are described in the Materials and Methods. The abbreviated species name of each sequence are: Rc, *R.communis* (castor); Sb, *Sorghum bicolor*; At, *Arabidopsis thaliana*; Os, *Oryza sativa* (rice); Stu, *S.tuberosum* (potato); Zm, *Z.mays* (maize); Gm, *G.max* (Soybean);Mcr, *M.crystallinum*; Synech, *Synechocystis sp*.PCC 6803(cyanobacteria).
